# Enhanced Sensitivity in Free Thyroxine Measurement Using Equilibrium Dialysis and LC-MS/MS Coupled with Offline Anion-Exchange Solid-Phase Extraction

**DOI:** 10.21627/v8rdvz16

**Published:** 2026-10-26

**Authors:** Julie A Ray, Kelly Doyle

**Affiliations:** ARUP Institute for Clinical and Experimental Pathology, Salt Lake City, UT, USA; ARUP Institute for Clinical and Experimental Pathology, Salt Lake City, UT, USA; Department of Pathology, University of Utah Health, and ARUP Laboratories, Salt Lake City, UT, USA

**Keywords:** Thyroid disease, free hormone, free thyroxine, equilibrium dialysis, solid-phase extraction, liquid chromatography–tandem mass spectrometry

## Abstract

**INTRODUCTION:** According to the free hormone hypothesis, only the unbound hormone fraction exerts biological effects. Diagnosis and monitoring of thyroid disorders often require the measurement of free thyroxine (fT4) by equilibrium dialysis, which is considered the gold-standard method for separating free hormones from biological samples. **OBJECTIVES:** Our objective was to reduce complexity and enhance the sensitivity of a liquid chromatography–tandem mass spectrometry method with online solid-phase extraction (SPE) for measuring fT4. **METHODS:** Serum aliquots of 200 µL were subjected to equilibrium dialysis. Post-dialysis, increases in analytical sensitivity and specificity were achieved using weak-anion-based SPE prior to liquid chromatographic separation with subsequent analyte detection by a high-sensitivity SCIEX Triple Quad™ 7500 mass spectrometer. **RESULTS:** Lower and upper limits of quantitation (LLOQ and ULOQ) were 0.1 and 10 ng/dL, respectively. Intra- and inter-day precision evaluated using four matrix-matched quality control samples (0.15, 2.09, 5.10, and 7.94 ng/dL) indicated <10% imprecision, showing excellent agreement with the previous validated method (slope = 1.022, *r* = 0.9927, bias = 4.01%, *n* = 123). Chromatograms exhibited a substantial improvement in the signal-to-noise ratio at the LLOQ. **CONCLUSION:** A complex liquid chromatographic separation with online trapping-column method was successfully transitioned to a simpler off-line SPE liquid chromatographic separation method. Incorporating SPE desalting and using the improved instrument sensitivity of the SCIEX Triple Quad™ 7500 mass spectrometer significantly improved the performance. A reduced injection volume allowed sample reanalysis, which is beneficial for pediatric samples. This method provides a superior analytical approach for clinical fT4 measurement.

## Non-Specialist Summary

We have developed a more accurate method to measure free thyroxine (fT4), a vital thyroid hormone for regulating body functions. Integrating an advanced purification step after equilibrium dialysis allows the test to measure low hormone concentrations more reliably than previous methods. This modification provides a substantial increase in the signal-to-noise ratio at the lower limit of quantitation (LLOQ), minimizing baseline interferences that cause errors. Because the test is more robust at the LLOQ, it is particularly effective at identifying thyroid issues in patients with very low hormone levels. Extensive testing confirms this method is reliable for more precise treatment decisions.

## 1. Introduction

An estimated 200 million people worldwide endure various forms of thyroid disease [1]. Thyroid diseases arise most commonly from iodine deficiency; however, in iodine-replete areas, autoimmune disorders are more frequent [2]. The risk for females developing hypo- or hyperthyroidism is approximately 5–10 times greater than that for males [3-5].

Evaluating thyroid function begins with measurements of thyroid stimulating hormone (TSH) because it is the most clinically sensitive biochemical test for detecting abnormal thyroid function [6, 7]. Abnormalities in TSH concentrations prompt subsequent free thyroxine (fT4) measurements to aid in differentiating primary from secondary causes of hyper- or hypothyroidism, monitoring therapeutic response to anti-thyroid medications [8,9], and following the progression of euthyroid sick syndrome [10-12]. Thyroxine (T4) is predominantly bound to thyroid binding globulin (TBG); a small portion is bound to transthyretin and albumin, with a minor portion bound to serum lipoproteins [13]. The percentage of free (unbound) T4 (fT4), which is considered to be the active or available fraction that can reach target tissues for biochemical activity, is very small (~0.01% of total T4). Although the concentration of fT4 is significantly lower than that of the bound fraction, it provides a more accurate assessment of thyroid status because transient fluctuations in total T4 are often influenced by changes in binding protein concentrations or binding capacity, which may occur during certain health conditions such as pregnancy [14], liver disease [15], severe illness [16], or when specific medications are used, including heparin [17], nonsteroidal anti-inflammatory drugs [18], and some anticonvulsants [19-21]. Very low plasma concentrations of fT4, in conjunction with narrow concentration distributions among healthy individuals, underscore the need for minimal imprecision and assay interference in an analytical method for this analyte.

Automated immunoassays used to measure thyroid hormones are known to be economical and rapid but also vulnerable to interferences that can confound interpretation and clinical correlation [22, 23]. Macro-TSH, thyroid hormone autoantibodies [24], anti-streptavidin antibodies [25], anti-ruthenium antibodies [26], biotin [27], and heterophilic antibodies [23] are potential interferences where immunoassays may be prone to positive or negative biases in thyroid analyte analysis. In addition, nonstandardized vendor immunoassays complicate result interpretation across different platforms [28, 29].

Physically separating protein-bound analyte from free analyte is a better alternative to immunoassay when interferences or protein binding abnormalities are suspected. Free hormones can be measured directly in serum by immunoassay or by separating the bound from the unbound fraction by direct methods such as ultrafiltration [30], equilibrium dialysis [31-34], or biocompatible solid-phase micro extraction (BioSPME) [35] coupled with analysis by isotope-dilution liquid chromatography–tandem mass spectrometry (LC-MS/MS). These sample preparation methods are designed to achieve both low detection limits of measurement and a high level of analyte specificity [36]. Although fast, ultrafiltration lacks accuracy because of operational variables, protein leakage, and nonspecific binding [37-41]. These factors often introduce bias and analyte loss, requiring careful management of pH, temperature, and centrifugal force during separation.

Equilibrium dialysis (ED) (coupled with LC-MS/MS) is the gold standard for measuring fT4 because it uses a semipermeable membrane to allow free hormones to reach a natural equilibrium between serum and buffer. This 20-h process at 37 °C ensures the final measurement accurately reflects the true free hormone levels without dilution [42]. However, results can still be skewed by adsorptive loss to the device [43, 44] or by interference from protein-binding drugs and fatty acids associated with heparin therapy [45].

In addition to sample preparation, the choice of detection technique also determines the precision and accuracy of fT4 measurements. Given that fT4 concentrations represent approximately 0.01% of the bound fraction, reliable detection of fT4 at low picogram per milliliter levels is essential. Several standard approaches to improve sensitivity of fT4 measurements may include increasing the sample volume for dialysis (Thermo Fisher Scientific Rapid Equilibrium Device) [46], using negative ionization mode to improve signal-to-noise (S/N) ratios [30], incorporating additives into the mobile phase, or increasing the injection volume by using LC-MS/MS in tandem with online solid-phase extraction (SPE) [31]. Although online SPE protocols may allow for simpler cleanup and reduced analyte loss, they also increase matrix load and the resultant ion suppression [47]. Researchers have investigated offline SPE and/or liquid–liquid extraction following the use of fast ED devices to reduce dialysis time and concentrate samples [48, 49]; however, the starting serum volume in these studies was large (1 mL).

In the present study, we demonstrate the enhanced analytical performance and workflow efficiency of an offline weak anion-exchange SPE protocol coupled with a next-generation mass spectrometer. This setup provides superior instrument sensitivity compared with an online trapping-column ED-LC-MS/MS protocol [31]. The method uses a 200 µL starting serum volume, and, to our knowledge, represents the first application of weak anion-exchange SPE for post-dialysis sample cleanup in fT4 analysis. Combining optimized sample preparation with improved instrument sensitivity enhanced performance at the lower limit of quantitation (LLOQ), increasing robustness and reducing repeat testing rates. Additionally, the simplified workflow reduced troubleshooting time, solvent consumption, and hazardous waste disposal. The resulting ED-LC-MS/MS method demonstrated robust fT4 quantitation with excellent accuracy, precision, and recovery rates as well as good sample reinjection capability and a significant increase in S/N ratios at the LLOQ.

## 2. Materials and Methods

### 2.1. Materials

Reagents and standards were prepared according to a previously described protocol [31]. Briefly, l-thyroxine (100 μg/mL solution of T4 in 0.1 N NH_3_ in methanol; Cerilliant, Round Rock, TX, USA) was used to prepare a master stock solution at 1 ng/mL in a mixture of 30% ammonium hydroxide (NH_4_OH) and methanol. The master stock solution was diluted with the methanol-based diluent to prepare eight intermediate calibration solutions with concentrations of 0.63, 3.13, 6.25, 12.5, 18.75, 25.0, 43.75, and 62.5 ng/dL. Each intermediate solution was further diluted with phosphate-buffered saline (PBS) containing HEPES, NaCl, Na_3_N, Na_3_PO_4_·12H_2_O, KCl, urea, CaCl_2_·2 H_2_O, and MgSO_4_·7H_2_O (Sigma-Aldrich, St Louis, MO, USA), as detailed previously [31], to prepare calibrators with concentrations of 0.1, 0.5, 1.0, 2.0. 3.0, 4.0, 7.0, and 10 ng/dL (1.29, 6.43, 12.87, 25.74, 38.61, 51.48, 90.09, and 128.7 pmol/L, respectively). The addition of 7.0 and 10.0 ng/dL calibrators expanded the analytical measurement range beyond the previous online trapping-column method. Stock internal standard l-thyroxine-^13^C_6_ (100 µg/mL; Cerilliant, Round Rock, TX, USA) was diluted to an intermediate concentration of 100 ng/mL. This standard solution was subsequently diluted with a mixture of methanol and phosphate-saline buffer (50 mmol/L Na_2_HPO4, 150 mmol/L NaCl, pH 10.9) to prepare a 2 ng/dL working solution. ED was performed using 96-well dialysis plates (Quik Prep 96-well Dispo Equilibrium Dialyzers, 10 kDa molecular weight cutoff, Harvard Apparatus, Holliston, MA, USA). Post-dialysis, SPE cleanup and concentration were evaluated on Strata-X polymeric weak anion-exchange SPE sorbent (Phenomenex, Torrance, CA) at both 10 and 30 mg sorbent masses. SPE elution solvent ranging between 5% and 15% NH_4_OH in methanol was also evaluated to optimize the peak area and S/N ratios.

### 2.2. Sample Preparation

Aliquots of 200 µL serum and quality controls were dialyzed for 20 ± 1 h against 200 µL of dialysis buffer at 37 °C [31]. Aliquots of 150 µL from calibrators (not subjected to dialysis), controls, and dialysates from patient samples were transferred into a 2.2 mL, 96-well collection plate (Agilent InfinityLab 96 deep-well collection plate). A working internal standard solution of l-thyroxine-^13^C_6_ (2 ng/dL, 150 µL) was added to each well, and the contents were mixed and centrifuged for 5 min at 1300g. The Phenomenex Strata-X weak anion-exchange SPE 10 and 30 mg beds were conditioned with 200 µL methanol, followed by equilibration with 200 µL water. The sample was loaded onto the SPE plates and allowed to sit for 5 min. SPE elution solution with various volumes of water:methanol was evaluated for optimum recovery of fT4. The fT4 was extracted from the SPE plate using a 2 × 200 µL optimized elution solution of 10% NH_4_OH in methanol and 1–3 psi of pressure. The plate was dried at 60 °C and reconstituted with 100 µL of 30% methanol in water. The contents were shaken for 5 min at 1300g and then placed on the autosampler for analysis.

#### 2.2.1. LC-MS/MS

Chromatographic separation was performed on a Kinetex C18 50 mm × 2.1 mm × 2.6 µm particle size high-pressure liquid chromatography (HPLC) column (Phenomenex) equipped with a Phenomenex Ultra security guard column (C18, 3 mm). The injection volume was 45 µL, and the column was maintained at 40 °C. Mobile phases A and B were 0.05% formic acid in water and a 1:1 (v/v) mixture of methanol and acetonitrile, respectively. Liquid chromatography separation was conducted with a flow rate of 0.5 mL/min and the following linear gradient profile: increased from 30% to 70% B over 2.5 min, maintained at 70% B for 0.5 min, increased to a 95% B flush from 3.0 to 4.0 min, and returned to 30% B over the final 1.5 min. The retention time for both T4 and ^13^C_6_-T4 was 2.6 min. The autosampler temperature was maintained at 10 °C and a mixture of methanol:water:Trifluoroacetic acid (80: 20: 0.1 v/v/v) was used as a needle wash. The nitrogen evaporator needles were regularly cleaned with a 1:1 methanol:water mixture during the sample drying step to prevent sample contamination. The instrument was kept clean by diverting flow into the mass spectrometer only between 1.5 and 4.0 min.

A SCIEX Triple Quad™ 7500 mass spectrometer (Framingham, MA, USA) was used in positive electrospray ionization mode. Quadrupoles Q1 and Q3 were tuned to unit resolution and optimized for maximum signal intensity. Collision-activated dissociation gas was set to 8. Q0 dissociation (Q0D) was used in place of declustering potential. Table 1 shows the parameters used for the SCIE X 7500.

**TABLE 1.** Mass spectrometer parameters.

Gas 1	70 psi
Gas 2	85 psi
Curtain gas	50 psi
Source temp.	600 °C
Spray voltage	2300 V

Compound ID	Q1 (Da)	Q3 (Da)	Dwell time (ms)	EP (V)	CE (V)	CXP (V)	Q0D (V)
fT4 quantitative	777.7	731.6	200	10	35	15	35
fT4 qualitative	777.7	604.7	200	10	55	15	45
^13^C_6_-T4 quantitative	783.8	737.8	200	10	35	15	35
^13^C_6_-T4 qualitative	783.8	610.7	200	10	55	15	45

Quantification of T4 was achieved using an eight-point calibration curve (0.1 to 10.0 ng/dL) and normalized to the ^13^C_6_ internal standard. Data acquisition and analysis were carried out using the OS software (version 3.1). The S/N ratio calculation and peak area integration were performed using the MQ 4 program in OS.

#### 2.2.2. Method performance

All samples underwent dialysis followed by SPE except the calibrators, which did not undergo dialysis. To establish the LLOQ, pH-controlled PBS spiked with 0.1 ng/dL T4 was subjected to SPE and analyzed in five replicates per day over 4 days (*n* = 20 total measurements). The predefined acceptance criteria for LLOQ were accuracy within ±20% of the target concentration and total imprecision (CV) ≤20%. Because patient specimens with fT4 concentrations near 0.1 ng/dL were not available in sufficient volume for repeated testing, a low concentration patient pool (~0.42 ng/dL) was used to assess precision at clinically relevant low concentrations. Ten replicate aliquots of the patient pool were subjected to ED followed by SPE and analyzed within a single analytical run. The analytical measurement range (AMR) was evaluated using PBS spiked with T4 at 0.1, 0.5, 1.0, 2.0, 3.0, 4.0, 7.0, and 10.0 ng/dL. Each concentration was analyzed in quadruplicate over 2 days. Quantification was performed using a linear calibration curve with 1/*x*^2^ weighting. Analytical precision was evaluated using five replicates of serum samples (quantity controls samples QC 1–4) over a period of 4 days. These QC samples (prepared independently from the calibrators, stable for 12 months) were prepared from Immuno Plus, Lyphocheck Levels 1 and 2 (Bio-Rad Laboratories, Hercules, California) appropriately spiked with T4 or diluted with vitamin D delipidized human serum (Golden West Diagnostics, LLC, Temecula, CA, USA) to reach the desired fT4 concentrations. The average concentrations of fT4 in these samples were 0.15, 2.09, 5.10, and 7.94 ng/dL (1.93, 26.89, 65.64, and 102.19 pmol/L, respectively). These QC samples placed in the front and end of every batch were evaluated according to Westgard QC rules prior to acceptance of patient data. To evaluate recovery during SPE, 20 low fT4 patient sample dialysates were spiked each day over a 2-day period (*n* = 40 total) with four concentrations of T4 spanning the AMR of the method (0.12, 1.2, 4, and 10 ng/dL). Calibrators, QCs, and patient samples were evaluated for acceptable chromatography, internal standard area accounts, and ion ratios.

A system suitability test (SST) sample comprising 4.9 pg/mL T4 and 9.8 pg/mL ^13^C_6_-T4 was prepared in the same solvent as the calibrators and was injected three times prior to each batch. The correct retention time for both the analyte and internal standard, as well as peak intensities consistent with typical operation, were noted.

Interference from small molecules (list provided in Supplementary Table S3) from hemoglobin (50, 150, 250, and 525 mg/dL), unconjugated bilirubin (1.7, 6.6, 16, and 30 mg/dL, from Sigma-Aldrich), and triglycerides (125, 250, 500, and 1000 mg/dL prepared from Sigma-Aldrich Intralipid 20, emulsion) were studied in three replicates of three serum samples with low, mid, and high concentrations of fT4 analyzed pre- and post-spike with the above interferences. Percent agreement with control was set to ±20%, and the CV% between replicates was set to ≤15%. Interference from triiodothyronine (T3) and reverse triiodothyronine (rT3) was also assessed.

Carryover was evaluated by injecting a low fT4 sample (0.14 ng/dL) after a high fT4 sample (>50 ng/dL) according to the procedure outlined in the carryover module of EP Evaluator (version 12.1). Retention-time acceptance criteria were set to be within 2.5% of the calibrator’s average retention time*.* Automated integration was preferred over manual integration.

Ion suppression was studied on 20 low-fT4-concentration samples (0.1 to 0.45 ng/dL) using a post–column infusion of T4 (200 ng/mL) into the LC flow. Chromatograms were examined for ≤25% change from the baseline because of suppression or enhancement at ±0.5 min of the retention time of the analyte. Ion ratio acceptance was set to ±30% of the average ratio of the calibrators*.*

Given that the total sample volume prior to the use of the SPE was 300 µL, sorbent masses of 10 and 30 mg were evaluated for peak areas and S/N ratio through analysis of three replicates of calibrator 1 and a low-fT4 patient dialysate (~1.2 ng/dL) for each sorbent mass. Peak areas and baseline noise were also evaluated in five low-fT4 patient pools for choosing the optimal percent base in the elution solvent (5, 8, 10, and 15% NH_4_OH in methanol).

Method comparison was performed using 123 specimens against a clinically validated method. The fT4 concentrations in these samples ranged from 0.15 to 44.7 ng/dL. These specimens were residual de-identified human specimens whose use was approved by the Institutional Review Board (IRB) of the University of Utah (IRB #7275). The specimen handling criteria and storage details remained the same as those previously reported [31]. Chromatograms, S/N ratios, and peak areas of fT4 at the low end of the AMR were compared between the methods.

## 3. Results

Method selectivity was confirmed by the strong retention of T4 on the Kinetex C18 column and its baseline separation from T3 and rT3 (Figure 1).

**Figure 1.**
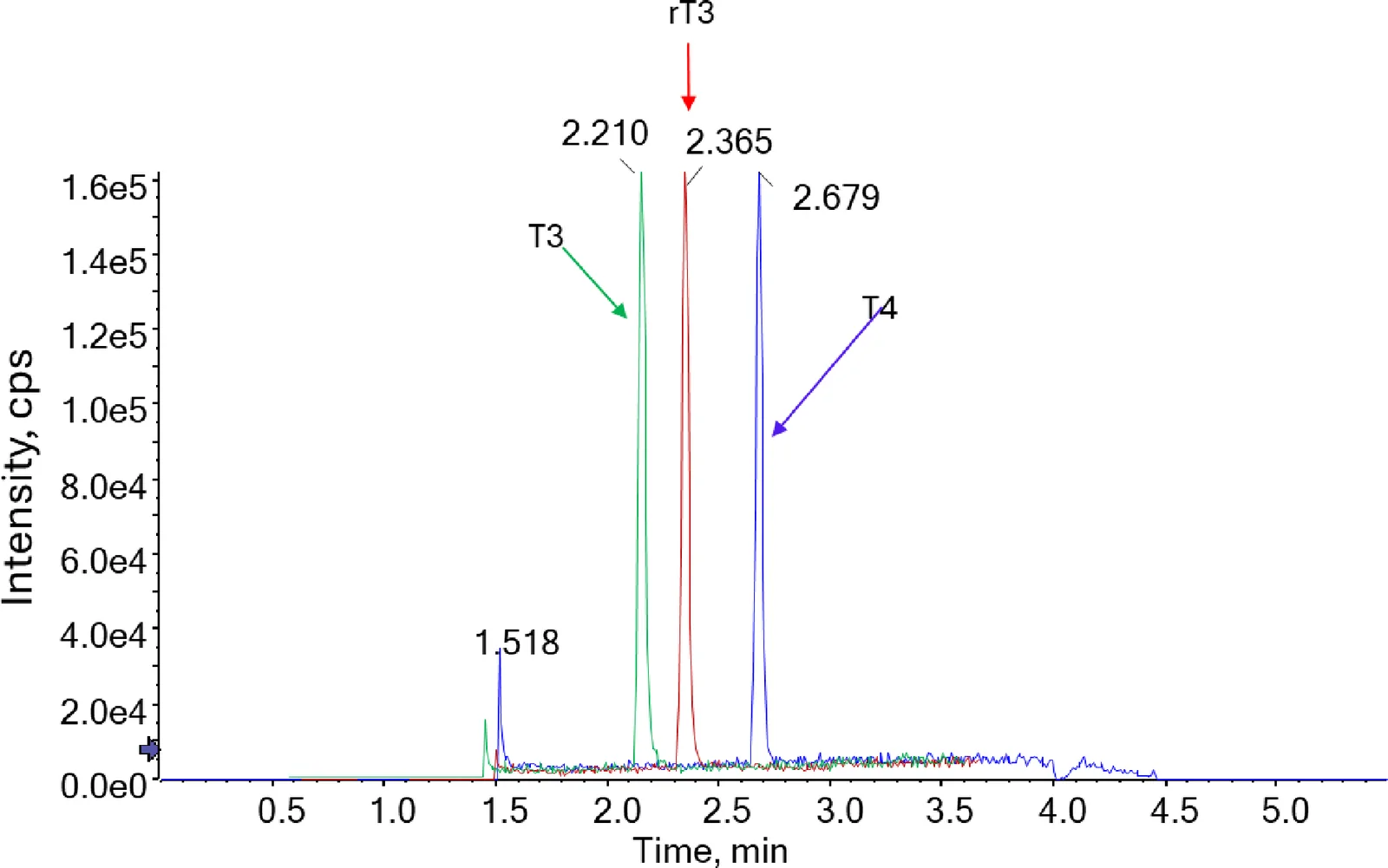
Chromatographic separation between T4, T3, and rT3.

The LLOQ was established using calibrator 1 (target concentration 0.1 ng/dL). Across 4 days of testing (*n* = 20 measurements), the mean concentration was 0.095 ng/dL (within-run and between-run/day CV%s were 3.34 and 0.62), with an accuracy of 95% relative to target. Both predefined acceptance criteria (accuracy within ±20% and CV ≤20%) were met; therefore, the LLOQ was established at 0.1 ng/dL (Supplementary Table S1).

To evaluate precision at low endogenous fT4 concentrations, a low patient pool with a concentration of 0.42 ng/dL was analyzed in 10 replicate preparations within a single run. The mean concentration was 0.408 ng/dL (SD = 0.014 ng/dL), corresponding to a within-run CV of 3.0% (Supplementary Table S2). Both calibrator 1 and low patient pool replicates were successfully auto integrated with an S/N >10 for the qualitative ion. There were no ion ratio failures. The AMR of the method determined by four replicates of the calibrators analyzed over 2 days showed excellent linearity (slope = 1.04, *r*^2^ = 1); the CV% at each level (ng/dL) was 0.1 (4%), 0.5 (4%), 1.0 (2%), 2.0 (3%), 3.0 (3%), 4.0 (3%), 7.0 (1%), and 10.0 (2%), with average accuracies ranging between 93% and 108% of the target concentration. The within-run, between-run, and total imprecision of the QC samples are shown in Table 2.

**TABLE 2.** Average concentration, within-run, between-run and total imprecision coefficient of variation (CV%) in QC samples (1–4).

Sample name	Avg concentration, ng/dL	Within-run CV (%)	Between-run CV (%)	Total CV (%)
QC 1	0.15	3.14	5.73	6.53
QC 2	2.09	3.08	3.68	4.80
QC 3	5.10	3.05	3.75	4.83
QC 4	7.94	3.37	5.04	6.06

Mean carryover was 0.03% for fT4, and there was no evidence of ion suppression, as observed near the retention time of the analyte for the quantitative or qualitative MRM ion channels.

No interference from hemoglobin, bilirubin, or triglycerides was observed at the evaluated concentrations with an average percent recovery of fT4 being 103%. T3 and rT3 were well separated from T4, eluting at 2.20 and 2.36 min, respectively (Supplementary Table S4).

The 10 mg sorbent bed demonstrated significantly improved performance, yielding more than twice the peak area and a more than 40% higher S/N ratio compared with the 30 mg bed (Table 3).

**TABLE 3.** Evaluation of SPE bed volume using patient sample with fT4 concentration of ~ 1.2 ng/dL.

Strata-X-AW sorbent mass	Average T4 peak area	S/N ratio
30 mg	4.21 × 10^5^	299
10 mg	9.53 × 10^5^	441

Washing the SPE wells with various volumes of water:methanol after sample loading did not significantly affect the S/N ratios or the drying time prior to elution. Although the T4 peak areas were similar between 10% and 15% conditions, 10% NH_4_OH in methanol yielded the highest S/N ratio (Supplementary Table S5).

Transitioning to the offline SPE method coupled with the AB Sciex Triple Quad™ 7500 mass spectrometer improved the quantitative S/N ratios by 5.8–19-fold, increased the qualitative ion S/N ratios by 3–17.3-fold, and doubled the peak areas at the 0.1 ng/dL threshold. The enhanced chromatographic resolution allowed for well-resolved peak integration. An overview of the modifications made to the online trapping-column method and the resultant performance enhancements are summarized in Table 4. Chromatographic comparisons for a low-concentration fT4 patient sample (0.72 ng/dL), a QC sample (0.11 ng/dL), and an SST sample (0.49 ng/dL T4, minimum T4 quant peak area 100,000 cps and S/N ratio >100) are shown in Figures 2a,b, 3a,b, and 4a,b, respectively. The differences in S/N ratios are listed in the figure legends.

**Figure 2.**
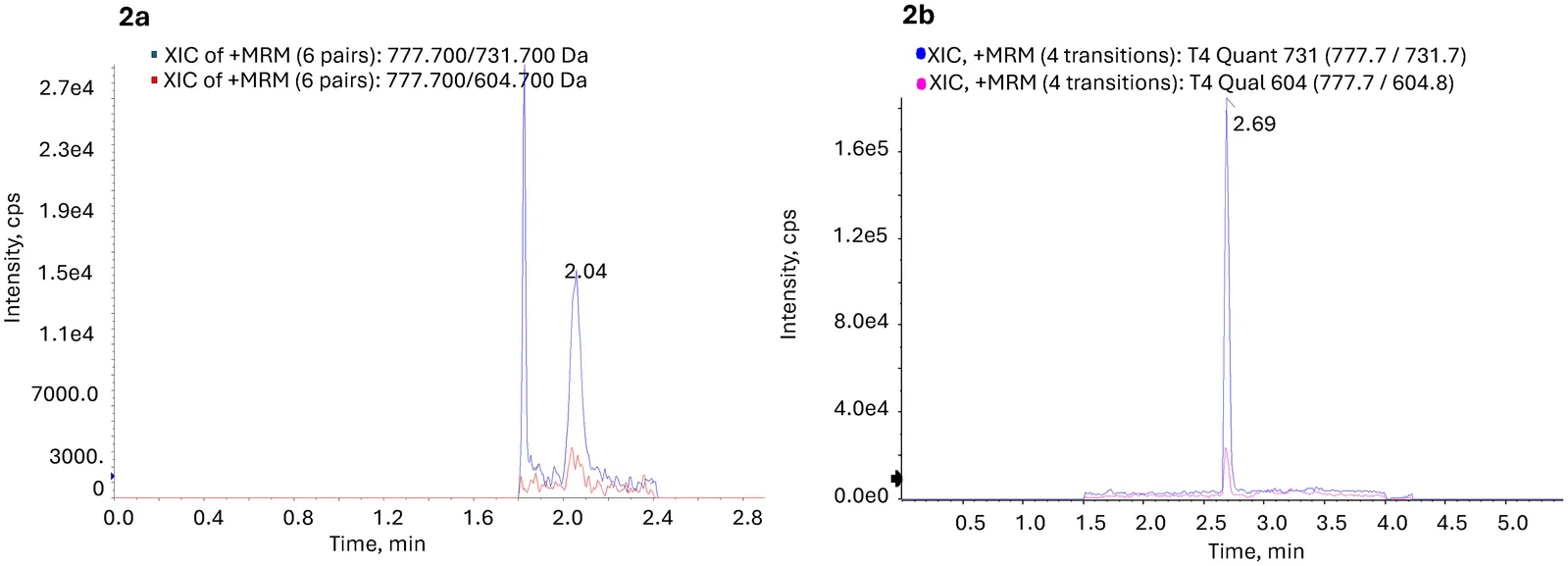
**a.** Patient sample with fT4 concentration of 0.72 ng/dL using the online trapping-column, 250 µL injection volume (blue trace representing fT4 quantitative ion and pink trace representing the qualitative ion). S/N ratio T4 quantitative = 13, qualitative = 8. **b.** Same specimen analyzed using the off-line SPE method with 45 µL injection volume (blue trace representing fT4 quantitative ion and pink trace representing the qualitative ion). S/N ratio T4 quantitative = 152, qualitative = 24.

**Figure 3.**
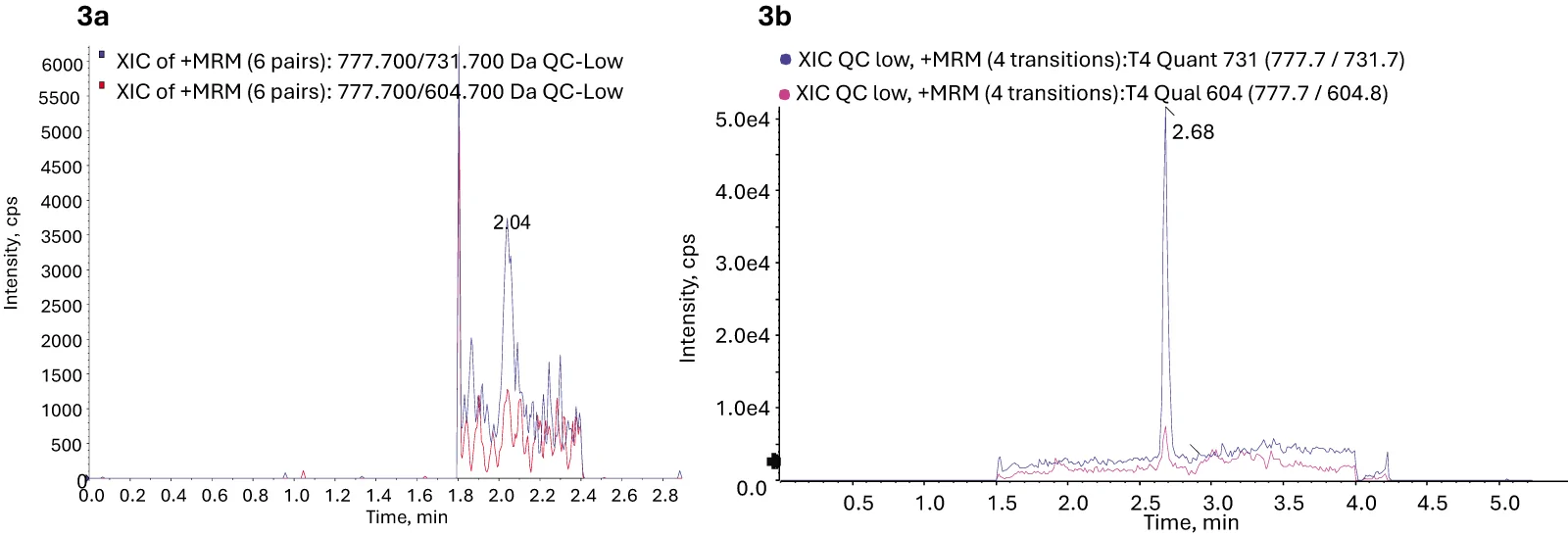
**a.** Quality control sample with fT4 concentration of 0.11 ng/dL using the online trapping-column, 250 µL injection volume (blue trace representing fT4 quantitative ion and pink trace representing the qualitative ion). S/N ratio T4 quantitative = 4, qualitative = NA. **b.** Quality control sample analyzed using the off-line SPE method with 45 µL injection volume (blue trace representing fT4 quantitative ion and pink trace representing the qualitative ion). S/N ratio T4 quantitative = 76, qualitative = 11.

**Figure 4.**
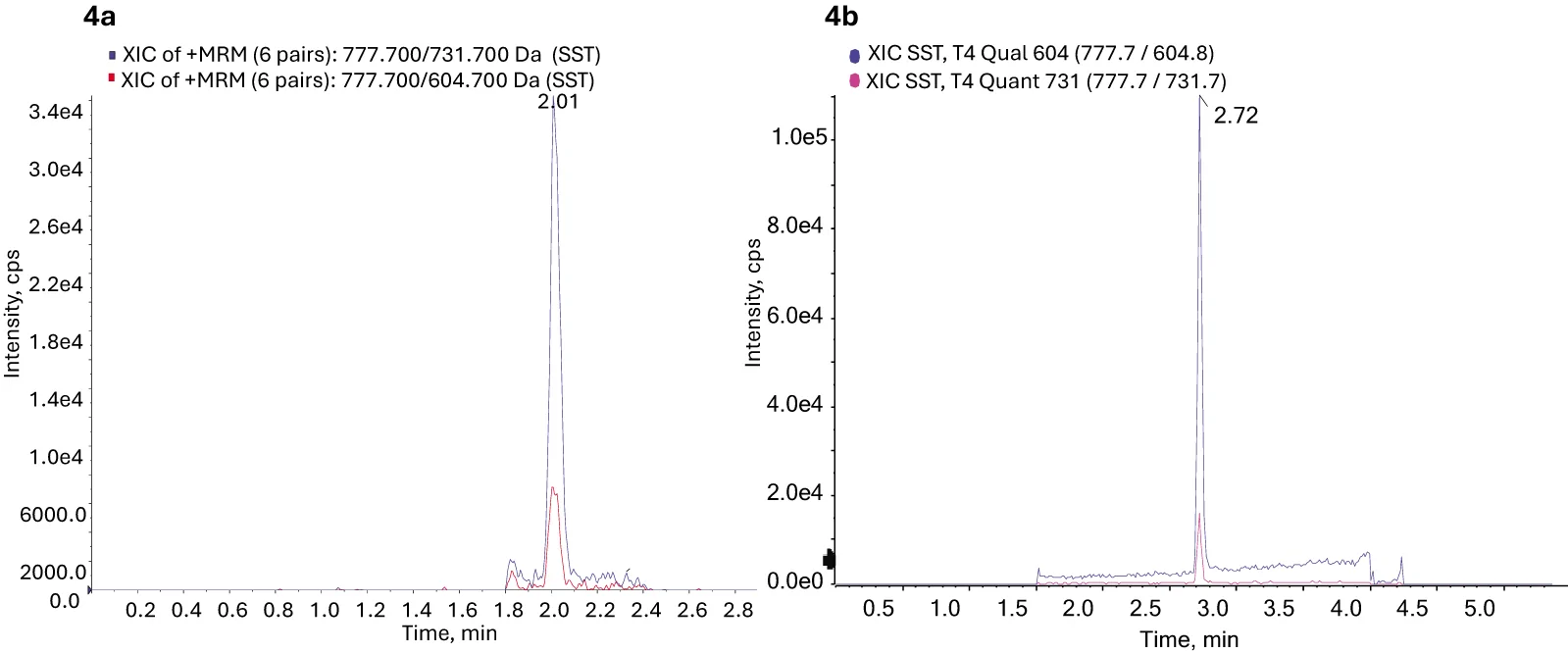
**a.** SST sample with fT4 concentration of 0.49 ng/dL using the online trapping-column method, 250 µL injection volume (blue trace representing fT4 quantitative ion and pink trace representing the qualitative ion). S/N ratio T4 quantitative = 20.4, qualitative = 6.4 **b.** SST sample with fT4 concentration of 0.49 ng/dL using the off-line SPE method with a 45 µL injection volume (blue trace representing fT4 quantitative ion and pink trace representing the qualitative ion). S/N ratio T4 quantitative = 118, qualitative = 111.

**TABLE 4.** Operational parameters and performance comparison between methods.

Parameters	Online trapping-column	Off-line SPE method
Instrumentation	AB SCIEX Triple Quad™ 5500	SCIEX Triple Quad™ 7500
Sample clean-up	Online C5, 4 × 2.0 mm	Off-line Strata-X polymeric weak anion exchange
LC column	Synergy Polar-RP 50 mm × 2 mm × 2 µm; Phenomenex	Kinetex C18 50 mm × 2.2 mm × 2.6 µm; Phenomenex
Mobile phase A	10 mM formic acid in water	0.05% formic acid in water
Mobile phase B	10 mM formic acid in methanol (loading column), 10 mM formic acid in acetonitrile (analytical column)	1:1 methanol:acetonitrile
Injection volume, µL	250	45
Run time, min	3.5	5.5
LLOQ, ng/dL	0.1	0.1
S/N ratio at QC 1 (0.11 ng/dL) quant/qual ion	4/NA	76/11

Results of Deming regression for evaluating spiked accuracy of SPE using 20 samples each on days 1 and 2 showed a slope of 0.9568 (95% CI: 0.9287 to 0.9850) and *Y*-intercept of 0.0078 (95% CI: –0.0965 to 0.1122). The average percent recovery was between 81% and 110% (Supplementary Table S6, Figure 2). A method comparison was performed between the online trapping-column method (SCIEX Triple Quad™ 5500 mass spectrometer) and the offline SPE-ED-LC-MS/MS method (SCIEX Triple Quad™ 7500 mass spectrometer). Deming results for 123 samples showed good comparison between the two methods, with a slope of 1.022 (95% CI: 0.9851 to 1.060) and *Y*-intercept of 0.0300 (95% CI: –0.0231 to 0.0832). Chromatograms showed no interference, and there were no ion ratio failures using the offline SPE method. Figure 5 shows the Deming regression scatter plot and Bland–Altman plot (where the *x*-axis represents the mean concentration measured by the online trapping-column method and offline SPE method and the *y*-axis represents the difference between the two; GraphPad Prism Ver 10.6.1). The mean bias was –0.077 ng/dL, and the 95% limits of agreement (LoAs) ranged from –0.318 ng/dL (95% CI: –0.356 to –0.281 ng/dL) to +0.165 ng/dL (95% CI: +0.128 to +0.203 ng/dL), confirming tight analytical agreement over the measurement range. A representative calibration curve is shown in Supplementary Figure S1.

**Figure 5.**
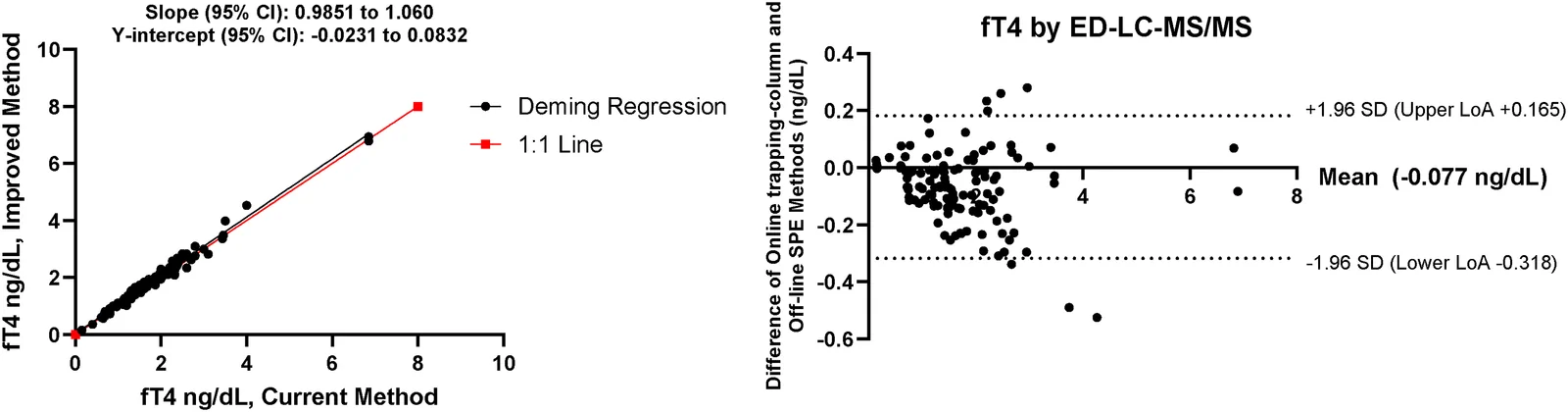
Deming regression and Bland–Altman scatter plot for method comparison between current and improved methods.

## 4. Discussion

The use of ED-LC-MS/MS for evaluating fT4 allows for greater specificity and accuracy for patients with binding-protein abnormalities or other substances that may interfere with fT4 immunoassays.

Previously reported ED-LC-MS/MS methods require large sample volumes and complex online trapping techniques while having poor S/N ratios at low analyte concentrations. With recent improvements to mass spectrometer sensitivity and offline SPE workflows, we sought to evaluate the measurement of fT4 using more sensitive instruments. Our goal was to reduce injection volume, eliminate online sample cleanup, and improve S/N ratios at low analyte concentrations. Following unsuccessful attempts to achieve the required outcomes of sensitivity and robustness using the Agilent 6470, 6475, and SCIEX Triple Quad™ 6500 mass spectrometers (data not shown), we pursued a multipronged approach to enhance analyte peak S/N ratios. This strategy involved concentrating and enriching the analyte through offline SPE (leveraging the amine, phenolic OH, and carboxyl functional groups of T4) while simultaneously exploiting the superior sensitivity and robustness of the SCIEX Triple Quad™ 7500 mass spectrometer.

The calibrators were prepared in the same matrix as the buffer used for dialysis, ensuring that the final matrix prior to SPE was the same for samples and calibrators. Because calibrators and patient sample dialysates lack protein during SPE, the absolute recovery, matrix effects, and extraction efficiencies were the same. To control any volume shifts or processing variations during dialysis, we added an internal standard to all of the calibrators, quality controls, and patient dialysates immediately post-dialysis, prior to SPE. The gold standard of ED for separating the free and bound T4 fractions was retained, and the offline SPE step provided crucial sample concentration and eliminated salts and buffers used during dialysis. Among the various SPE phases evaluated for increased recovery, reverse-phase weak anion-exchange SPE (di-amino) exhibited the best recovery and accuracy. Optimizing the SPE protocol demonstrated that reducing the Strata-X-AW sorbent bed mass from 30 to 10 mg significantly improved the extraction efficiency. We attributed this improvement to minimized nonspecific retention of matrix interferences on the smaller sorbent bed. Ultimately, a 2.2-fold increase in peak areas and a significant increase in S/N ratio supported a more reliable and robust LLOQ.

Because of the high selectivity of the proposed method, no post-loading wash was required. Optimal fT4 elution was achieved using 10% NH_4_OH in methanol. Lower concentrations (5% and 8%) caused incomplete active-site neutralization and poorer recoveries, whereas a 15% solution sharply reduced the S/N ratio. The elevated baseline noise from the 15% solution likely stemmed from coelution of strongly bound matrix interferences (Supplementary Material Table S1).

The improved offline SPE method uses a much lower starting serum volume than other available methods based on similar technology. Utilizing offline SPE in conjunction with a more sensitive mass spectrometer dramatically reduced injection volumes, repeat sample preparation time, and technical troubleshooting typically associated with online trapping-column LC-MS/MS. Consistently high peak areas, high S/N ratios, and operational simplicity made the proposed method a more practical solution for fT4 quantitation.

These analytical improvements translated into significant clinical benefits. We observed improved imprecision at low concentrations, reduced repeat dialysis within the laboratory because of decreased injection volumes, and an 18% reduction in requests for recollection because this method consumes less patient sample.

Furthermore, because the reduced injection volume allows for multiple injections, samples can be easily re-injected and re-analyzed after instrument interruptions. This process reduced the need for repeat 96-well plate preparations by 34%, contrasting sharply with the online trapping-column method, where limited sample volume hindered troubleshooting and required time-intensive re-dialysis (20 ± 1 h). Post ED, off-line SPE enrichment combined with superior mass spectrometric detection substantially increased the S/N ratio and doubled peak areas at the LLOQ. This advancement was especially advantageous at the critical low-end threshold, offering clinical laboratories a robust and cost-effective tool for low-level fT4 quantification.

## 5. Conclusion

Automated immunoassay methods used for the measurement of fT4 remain susceptible to matrix-induced limitations. Although ED followed by LC-MS/MS offers superior sensitivity and specificity, maximizing throughput requires further optimization. This study describes an enhanced analytical platform that substantially increases robustness of low-end sensitivity for measuring serum fT4. By integrating an optimized post-dialysis offline SPE protocol with an advanced mass spectrometry platform, the system yielded higher S/N ratios and doubled peak areas at the LLOQ. These performance gains were achieved while simplifying chromatography from an online trapping-column to a streamlined configuration. Consequently, this modification reduced injection volumes fivefold, stabilized baseline peak integration, reduced repeat testing, and enhanced lower-end reporting accuracy.

## Supplementary Material

Supplementary Material

## Data Availability

Summary data supporting the validated method are included in the article and Supplementary Material. The underlying de-identified validation and method comparison data supporting the LLOQ, analytical measurement range, precision, recovery, interference, carryover, ion suppression, SPE optimization, and method comparison analyses are available upon request from the corresponding author at julie.ray@aruplab.com, subject to institutional restrictions. Data derived from residual anonymized human donor samples were used under University of Utah IRB approval #7275. Individual-level–de-identified validation metrics and method comparison data can be shared upon reasonable request subject to institutional data governance review. Only aggregate summaries are publicly available with the article and supplementary files to maintain compliance with the institution regarding residual clinical specimens. No custom code or analysis scripts were used in this study. Data acquisition and analysis used SCIEX OS software version 3.1; carryover and method comparison analyses used EP Evaluator versions 12.1 and 12.3.0.2; and Bland–Altman plotting used GraphPad Prism version 10.6.1.

## Abbreviations

AMR: Analytical measurement range
BioSPME: Biocompatible solid-phase microextraction
ED: Equilibrium dialysis
fT3: Free triiodothyronine
fT4: Free thyroxine
LC-MS/MS: liquid chromatography–tandem mass spectrometry
LLOQ: Lower limit of quantitation
QC: Quality control
SPE: Solid-phase extraction
TBG: Thyroid binding globulin
ULOQ: Upper limit of quantitation

## Supplementary Material

Supplemental Material Available at https://doi.org/10.21627/v8rdvz16
