# Supplementary material for: Enhanced Sensitivity in Free Thyroxine Measurement Using Equilibrium Dialysis and LC-MS/MS Coupled with Offline Anion-Exchange Solid-Phase Extraction
Source: J Mass Spectrom Adv Clin Lab. 2026 Oct 26;39(1):1-17. doi: 10.21627/v8rdvz16 (PMC13756137; doi:10.21627/v8rdvz16)
Supplement: Supplementary file 1 [file jmsacl-39-1-v8rdvz16-s001.docx]

**Supplementary Material**

**Enhanced Sensitivity in Free Thyroxine Measurement using Equilibrium Dialysis and LC-MS/MS coupled with Off-line Anion Exchange Solid Phase Extraction**

Julie A Ray^1^, Kelly Doyle^1,2^

^1^ARUP Institute for Clinical and Experimental Pathology, Salt Lake City, UT, USA

^2^Department of Pathology, University of Utah Health, and ARUP Laboratories, Salt Lake City, UT, USA

Figure 1. Representative calibration curve.

**Table 1a.** Within-run, between-run/day and total CV % for establishing LLOQ using calibrator 1.

| Sample Name | fT4 ng/dL, Day 1 | fT4 ng/dL, Day 2 | fT4 ng/dL, Day 3 | fT4 ng/dL, Day 4 | Within-run CV % | Between-run/day CV % | Total CV % | Mean fT4 ng/dL | % Accuracy |
| --- | --- | --- | --- | --- | --- | --- | --- | --- | --- |
| Calibrator 1 -1 | 0.100 | 0.096 | 0.094 | 0.099 | 3.34 | 0.62 | 3.40 | 0.0952 | 95% |
| Calibrator 1 -2 | 0.093 | 0.094 | 0.090 | 0.095 |  |  |  |  |  |
| Calibrator 1 -3 | 0.096 | 0.094 | 0.095 | 0.099 |  |  |  |  |  |
| Calibrator 1 -4 | 0.091 | 0.094 | 0.098 | 0.097 |  |  |  |  |  |
| Calibrator 1 -5 | 0.101 | 0.096 | 0.089 | 0.093 |  |  |  |  |  |

**Table 1b**. Within-run, between-run/day and total CV % for evaluating precision of LLOQ using low patient pool.

| Sample Name | fT4, ng/dL | Average fT4, ng/dL | SD | % CV |
| --- | --- | --- | --- | --- |
| Sens Low Pt pool 1-1 | 0.434 | 0.422 | 0.014 | 3% |
| Sens Low Pt pool 1-2 | 0.410 |  |  |  |
| Sens Low Pt pool 1-3 | 0.432 |  |  |  |
| Sens Low Pt pool 1-4 | 0.430 |  |  |  |
| Sens Low Pt pool 1-5 | 0.407 |  |  |  |
| Sens Low Pt pool 1-6 | 0.408 |  |  |  |
| Sens Low Pt pool 1-7 | 0.448 |  |  |  |
| Sens Low Pt pool 1-8 | 0.428 |  |  |  |
| Sens Low Pt pool 1-9 | 0.409 |  |  |  |
| Sens Low Pt pool 1-10 | 0.414 |  |  |  |

**Table 2a**. List of interferences evaluated.

| **Small Molecules** | **Interference Mix 1** | **Interference Mix 2** |
| --- | --- | --- |
| Prednisone | (-)-Cotinine (1000 µg/mL) | Fentanyl |
| Prednisolone | (-)-Nicotine (1000 µg/mL) | Norfentanyl oxalate |
| Dexamethasone | Acetaminophen (1000 µg/mL) | Naloxone |
| 11-deoxycortisol | Caffeine (1000 µg/mL) | Morphine |
| Corticosterone | Ibuprofen (1000 µg/mL) | Zolpidem |
| DHEA | Naproxen (1000 µg/mL) | Alpha-Hydroxymidazolam |
| Estradiol | Phentermine (100 µg/mL) | Midazolam |
| 21-deoxy cortisol | R,R(-)-Pseudoephedrine, List 1 (100 µg/mL) | Codeine, 1mg/mL |
| Testosterone | Chlorpheniramine Maleate (100 µg/mL) | Oxymorphone |
| Progesterone |  | Oxycodone |
| Aldosterone |  | Buprenorphine |
| Cortisol |  | Meperidine |
| Cortisone |  | Methylphenidate |
| rT3 |  | (±)-Amphetamine |
| T3 |  | Ritalinic Acid |
|  |  | Tapentadol HCl |
|  |  | Norhydrocodone HCl |
|  |  | (±)-3,4-Methylenedioxyamphetamine |
|  |  | Methylenedioxyethylamphetamine |
|  |  | (±)-3,4-Methylenedioxymethamphetamine |
|  |  | Norbuprenorphine |
|  |  | (±)-Methamphetamine |
|  |  | 6-acetylmorphine |
|  |  | Alpha-Hydroxyalprazolam |
|  |  | Hydrocodone |
|  |  | Hydromorphone |
|  |  | Clonazepam |
|  |  | 7-Aminoclonazepam |
|  |  | Alprazolam |
|  |  | Oxazepam |
|  |  | Temazepam |
|  |  | Diazepam |
|  |  | Nordiazepam |
|  |  | Lorazepam |
|  |  | Phentermine |
|  |  | Noroxycodone |
|  |  | Noroxymorphone hydrochloride |
|  |  | Normeperidine |
|  |  | Zolpidem Phenyl-4-carboxylic acid |

**Table 2b.** Accuracy of low and mid pool spiked with hemoglobin (Hb) , triglycerides (Tg), and bilirubin (Bili).

| Sample Name | fT4 ng/dL | Average fT4, ng/dL | SD | % CV | % Accuracy of spiked samples |
| --- | --- | --- | --- | --- | --- |
| No spike Low pool 1-1 | 0.588 | 0.587 | 0.015 | 3% |  |
| No spike Low pool 1-2 | 0.601 |  |  |  |  |
| No spike Low pool 1-3 | 0.571 |  |  |  |  |
| Low pool Hb Control Spike 1-1 | 0.616 | 0.581 | 0.032 | 5% | 99% |
| Low pool Hb Control Spike 1-2 | 0.554 |  |  |  |  |
| Low pool Hb Control Spike 1-3 | 0.574 |  |  |  |  |
| Low pool Hb Spike 1-1 | 0.666 | 0.614 | 0.047 | 8% | 105% |
| Low pool Hb Spike 1-2 | 0.575 |  |  |  |  |
| Low pool Hb Spike 1-3 | 0.600 |  |  |  |  |
| Low pool Tg Control Spike 1-1 | 0.598 | 0.578 | 0.019 | 3% | 99% |
| Low pool Tg Control Spike 1-2 | 0.560 |  |  |  |  |
| Low pool Tg Control Spike 1-3 | 0.577 |  |  |  |  |
| Low pool Tg Spike 1-1 | 0.553 | 0.570 | 0.024 | 4% | 93% |
| Low pool Tg Spike 1-2 | 0.559 |  |  |  |  |
| Low pool Tg Spike 1-3 | 0.597 |  |  |  |  |
| Low pool Bili Control Spike 1-1 | 0.543 | 0.549 | 0.010 | 2% | 95% |
| Low pool Bili Control Spike 1-2 | 0.543 |  |  |  |  |
| Low pool Bili Control Spike 1-3 | 0.560 |  |  |  |  |
| Low pool Bili Spike 1-1 | 0.546 | 0.534 | 0.013 | 2% | 94% |
| Low pool Bili Spike 1-2 | 0.536 |  |  |  |  |
| Low pool Bili Spike 1-3 | 0.521 |  |  |  |  |
| No spike Mid pool 1-1 | 1.441 | 1.426 | 0.023 | 2% |  |
| No spike Mid pool 1-2 | 1.437 |  |  |  |  |
| No spike Mid pool 1-3 | 1.400 |  |  |  |  |
| Mid pool Hb Control Spike 1-1 | 1.479 | 1.470 | 0.023 | 2% | 103% |
| Mid pool Hb Control Spike 1-2 | 1.443 |  |  |  |  |
| Mid pool Hb Control Spike 1-3 | 1.487 |  |  |  |  |
| Mid pool Hb Spike 1-1 | 1.412 | 1.399 | 0.016 | 1% | 98% |
| Mid pool Hb Spike 1-2 | 1.404 |  |  |  |  |
| Mid pool Hb Spike 1-3 | 1.382 |  |  |  |  |
| Mid pool Tg Control Spike 1-1 | 1.454 | 1.436 | 0.025 | 2% | 98% |
| Mid pool Tg Control Spike 1-2 | 1.446 |  |  |  |  |
| Mid pool Tg Control Spike 1-3 | 1.407 |  |  |  |  |
| Mid pool Tg Spike 1-1 | 1.467 | 1.379 | 0.106 | 8% | 99% |
| Mid pool Tg Spike 1-2 | 1.410 |  |  |  |  |
| Mid pool Tg Spike 1-3 | 1.261 |  |  |  |  |
| Mid pool Bili Control Spike 1-1 | 1.294 | 1.294 | 0.021 | 2% | 90% |
| Mid pool Bili Control Spike 1-2 | 1.273 |  |  |  |  |
| Mid pool Bili Control Spike 1-3 | 1.315 |  |  |  |  |
| Mid pool Bili Spike 1-1 | 1.376 | 1.365 | 0.010 | 1% | 99% |
| Mid pool Bili Spike 1-2 | 1.356 |  |  |  |  |
| Mid pool Bili Spike 1-3 | 1.363 |  |  |  |  |

**Table 3.** Evaluating T4 peak areas and S/N ratios using various percentages of NH_4_OH in methanol.

| **NH_4_OH in Methanol (%)** | **T4 peak area** | **S/N ratio** |
| --- | --- | --- |
| 5 | 6.90E+04 | 59.7 |
| 8 | 6.44E+04 | 78.4 |
| 10 | 7.19E+04 | 93.4 |
| 15 | 7.10E+04 | 73.5 |

**Table 4**. % Recovery of spiked samples during SPE.

**Day 1**

| Sample Name (unit for spiked amount, ng/dL) | Expected fT4 ng/dL | Observed fT4 ng/dL | % Recovery |
| --- | --- | --- | --- |
| No spike Sp 1 |  | 1.298 |  |
| Spike 0.1 Sp 1 | 1.398 | 1.162 | 83% |
| No spike Sp 2 |  | 0.925 |  |
| Spike 0.1 Sp 2 | 1.025 | 0.909 | 89% |
| No spike Sp 3 |  | 0.613 |  |
| Spike 0.1 Sp 3 | 0.713 | 0.665 | 93% |
| No spike Sp 4 |  | 0.716 |  |
| Spike 0.1 Sp 4 | 0.816 | 0.74 | 91% |
| No spike Sp 5 |  | 0.58 |  |
| Spike 0.1 Sp 5 | 0.68 | 0.621 | 91% |
| No spike Sp 6 |  | 0.99 |  |
| Spike 0.5 Sp 6 | 1.49 | 1.206 | 81% |
| No spike Sp 7 |  | 0.898 |  |
| Spike 0.5 Sp 7 | 1.398 | 1.194 | 85% |
| No spike Sp 8 |  | 1.263 |  |
| Spike 0.5 Sp 8 | 1.763 | 1.491 | 85% |
| No spike Sp 9 |  | 0.683 |  |
| Spike 0.5 Sp 9 | 1.183 | 1.046 | 88% |
| No spike Sp 10 |  | 0.767 |  |
| Spike 0.5 Sp 10 | 1.267 | 1.125 | 89% |
| No spike Sp 11 |  | 1.715 |  |
| Spike 1.0 Sp 11 | 2.715 | 2.365 | 87% |
| No spike Sp 12 |  | 1.015 |  |
| Spike 1.0 Sp 12 | 2.015 | 1.967 | 98% |
| No spike Sp 13 |  | 1.704 |  |
| Spike 1.0 Sp 13 | 2.704 | 2.44 | 90% |
| No spike Sp 14 |  | 2.464 |  |
| Spike 1.0 Sp 14 | 3.464 | 3.18 | 92% |
| No spike Sp 15 |  | 1.482 |  |
| Spike 1.0 Sp 15 | 2.482 | 2.261 | 91% |
| No spike Sp 16 |  | 1.227 |  |
| Spike 2.0 Sp 16 | 3.227 | 3.469 | 107% |
| No spike Sp 17 |  | 0.988 |  |
| Spike 2.0 Sp 17 | 2.988 | 2.876 | 96% |
| No spike Sp 18 |  | 1.461 |  |
| Spike 2.0 Sp 18 | 3.461 | 3.508 | 101% |
| No spike Sp 19 |  | 1.816 |  |
| Spike 2.0 Sp 19 | 3.816 | 4.092 | 107% |
| No spike Sp 20 |  | 1.705 |  |
| Spike 2.0 Sp 20 | 3.705 | 3.7 | 100% |

**Day 2**

| Sample Name (unit for spiked amount, ng/dL) | Expected fT4 ng/dL | Observed fT4 ng/dL | % Recovery |
| --- | --- | --- | --- |
| No spike Sp 1 |  | 1.308 |  |
| Spike 2.6 Sp 1 | 3.975 | 3.517 | 88% |
| No spike Sp 2 |  | 1.25 |  |
| Spike 2.6 Sp 2 | 3.917 | 3.429 | 88% |
| No spike Sp 3 |  | 0.654 |  |
| Spike 2.6 Sp 3 | 3.321 | 3.227 | 97% |
| No spike Sp 4 |  | 0.575 |  |
| Spike 2.6 Sp 4 | 3.242 | 3.156 | 97% |
| No spike Sp 5 |  | 0.647 |  |
| Spike 2.6 Sp 5 | 3.314 | 3.282 | 99% |
| No spike Sp 6 |  | 0.8 |  |
| Spike 4.0 Sp 6 | 4.8 | 4.789 | 100% |
| No spike Sp 7 |  | 0.63 |  |
| Spike 4.0 Sp 7 | 4.63 | 4.552 | 98% |
| No spike Sp 8 |  | 0.197 |  |
| Spike 4.0 Sp 8 | 4.197 | 4.386 | 105% |
| No spike Sp 9 |  | 0.831 |  |
| Spike 4.0 Sp 9 | 4.831 | 4.883 | 101% |
| No spike Sp 10 |  | 0.571 |  |
| Spike 4.0 Sp 10 | 4.571 | 4.713 | 103% |
| No spike Sp 11 |  | 2.695 |  |
| Spike 6.0 Sp 11 | 8.695 | 8.404 | 97% |
| No spike Sp 12 |  | 2.73 |  |
| Spike 6.0 Sp 12 | 8.73 | 8.449 | 97% |
| No spike Sp 13 |  | 2.299 |  |
| Spike 6.0 Sp 13 | 8.299 | 8.086 | 97% |
| No spike Sp 14 |  | 1.5 |  |
| Spike 6.0 Sp 14 | 7.5 | 8.251 | 110% |
| No spike Sp 15 |  | 2.258 |  |
| Spike 6.0 Sp 15 | 8.258 | 7.446 | 90% |
| No spike Sp 16 |  | 1.688 |  |
| Spike 10.0 Sp 16 | 11.688 | 11.101 | 95% |
| No spike Sp 17 |  | 1.11 |  |
| Spike 10.0 Sp 17 | 11.11 | 10.408 | 94% |
| No spike Sp 18 |  | 1.634 |  |
| Spike 10.0 Sp 18 | 11.634 | 10.808 | 93% |
| No spike Sp 19 |  | 1.871 |  |
| Spike 10.0 Sp 19 | 11.871 | 11.333 | 95% |
| No spike Sp 20 |  | 1.931 |  |
| Spike 10.0 Sp 20 | 11.931 | 11.01 | 92% |

**Figure 2.** Deming regression for evaluating spiked accuracy of the off-line SPE method.
